# Comparison of the effect of Everolimus, Prednisolone, and a combination of both on experimentally induced peritoneal adhesions in rats

**DOI:** 10.1038/s41598-024-61620-3

**Published:** 2024-05-14

**Authors:** Kourosh Kazemi, Kamran Jamshidi, Reyhaneh Naseri, Reza Shahriarirad, Alireza Shamsaeefar, Ahmad Hosseinzadeh

**Affiliations:** 1 Shiraz Transplant Center, Abu Ali Sina Hospital, Shiraz University of Medical Sciences, Shiraz, Iran; 2 Department of Surgery, Shiraz University of Medical Sciences, Shiraz, Iran; 3 Student Research Committee, School of Medicine, Shiraz University of Medical Sciences, Shiraz, Iran; 4 Thoracic and Vascular Surgery Research Center, Shiraz University of Medical Science, Shiraz, Iran; 5 School of Medicine, Shiraz University of Medical Sciences, Shiraz, Iran

**Keywords:** Peritoneal adhesion, Everolimus, Adhesion band, Animal model, Rats, Prednisolone, Gastrointestinal models, Preclinical research

## Abstract

Postoperative intra-abdominal adhesions represent a significant post-surgical problem. Its complications can cause a considerable clinical and cost burden. Herein, our study aimed to investigate the effect of Everolimus on peritoneal adhesion formation after inducing adhesions in rats. In this experimental study, adhesion bands were induced by intraperitoneal injection of 3 ml of 10% sterile talc solution in 64 male albino rats. The first group served as the control group. The second one received oral Prednisolone (1 mg/kg/day), the third received Everolimus (0.1 mg/kg/day), and group four received both drugs with similar dosages for four consecutive weeks. The formation of adhesion bands was qualitatively graded according to the Nair classification. The rats in the control group had extensive adhesions between the abdominal wall and the organs. Regarding substantial adhesion formation, 50% (8/16) of animals in the control group had substantial adhesions, while this rate in the groups receiving Prednisolone, Everolimus, and combination treatment was 31%, 31%, and 31%, respectively. Also, 68.75% (5/11) of the Prednisolone recipients had insubstantial adhesions, the same as Everolimus recipients, while in the combination group, 66.66% (10/15) rats had insubstantial adhesions. Everolimus demonstrated satisfactory results in reducing the rates of induced peritoneal adhesion in an experimental model, similar to Prednisolone and superior to a combination regime.

## Introduction

Intra-abdominal peritoneal adhesions are fibrotic pathological bonds that cause a significant surgical challenge in all abdominal surgeries^1,2^. They are usually formed between the omentum, loops of the bowel, and the abdominal wall^1^. The eccentric wound healing process causes the formation of intra-abdominal adhesions; thus, mesothelial damages like surgical traumas, bacterial infections, chemical irritations, and inflammations can produce these bonds^3–6^. Notwithstanding that the complications of adhesions can appear decades after the procedure, imposing a challenge of considerable magnitude for clinicians^7^. The most frequent complications include meteorism, irregular bowel movements, acute small bowel obstruction, acute intestinal perforation, chronic abdominal and pelvic pain, digestive disorders, dyspareunia, secondary infertility in females, and urologic dysfunction^2,8,9^.

Postoperative adhesions also pose a considerable health problem with notable effects on the quality of life and profound implication on financial health care expenses^2,10^. A population-based study estimated an annual direct and indirect cost of £2 million regarding bowel obstruction secondary to postoperative adhesions^11^. Another study reported that adhesive bowel obstruction may annually cause 2330 hospital admissions and a direct cost of about US$13 million^12^; Therefore, evaluating mechanisms for decreasing this significant financial burden to healthcare providers is essential.

The incidence of postoperative adhesions is increasing worldwide based on the increased frequency of abdominal surgery^13^. Various studies over the decades have aimed to develop possible solutions to reduce the rate of postoperative peritoneal adhesion through different methods. Surgical approaches, such as utilizing mechanical barriers to prevent contact of manipulated organ surfaces^14^ or pharmacological prophylactic anti-adhesiogenic agents^15^, have been proposed; however, limited effectiveness in obliterating the risk of adhesion formation has been reported^8^. Among pharmaceutical agents, Prednisolone as a corticosteroid inhibits fibroblast proliferation^16^ and targets the inflammatory components of the pathogenesis of adhesion formation^17^.

As surgeons in transplant centers, we observed a lower incidence of adhesion bonds after liver transplant during re-exploration. We hypothesized that steroid-free immunosuppressants such as Everolimus could reduce peritoneal adhesion bands. In line with our proposition, in a retrospective study in 2001, among 4001 post-liver transplantation patients, 48 patients (1.2%) developed postoperative bowel obstruction, of which 19 (0.5%) were secondary to postoperative peritoneal adhesions^18^.

Based on our hypothesis and information available in the literature, we designed this study to investigate the effectiveness of orally administered Everolimus, Prednisolone, and a combination of them in preventing or reducing intraperitoneal adhesion bands in rat models.

## Methodology

### Study design

This study was a randomized and exploratory animal study. The current research was conducted at the Center of Comparative and Experimental Medicine, Shiraz University of Medical Sciences, Shiraz, Iran, and the study’s protocol followed the principles of the guideline of the Ministry of Health and Education of Medicine of Iran for care and use of laboratory animals, with ethical approval from the Institutional Animal Care and Use Committee of Research Center of Shiraz University of Medical Sciences (Ethical code: IR.SUMS.MED.REC.1395.S221). The sample size was assigned based on similar previous reports, and the minimum number of subjects needed, along with taking into account the risk of drop-out/mortality^19–23^. All attempts to minimize animal distress and to employ only the number of animals essential to attain reliable results were made.

In this study, 64 male albino rats weighing 200–250 gr and aged between 8 and 10 weeks were obtained from the animal house of Shiraz university of medical sciences, Shiraz, Iran. Their health status was monitored in standard and controlled circumstances in the animal laboratory. All rats were randomized into four equal groups of 16 and kept in similar metal shelves with 55 ± 5% relative humidity, a 12/12-h light/dark cycle, and 22 °C ± 2 °C temperature. All animals had free access to water and food ad libitum and were well-nourished.

### Adhesion induction

Rats were fasted overnight before adhesion induction but with free access to water. To induce peritoneal adhesions, all rats received a single intraperitoneal injection of 3 mL of a 10% sterile talc solution [hydrated magnesium silicate, Mg_3_Si_4_O_10_(OH)_2_], (Shimico, Tehran, Iran) as previously reported^24,25^.

### Animal grouping

Among the four groups in our study, the first group served as the control group and received no drug; the second group was given 1 mg/ kg oral prednisolone (as 5 mg tablets made by Iranhormone factory, Iran) daily in the morning using long metal gavage. The third group received 0.1 mg/kg oral Everlimous (Wyeth Pharmaceuticals, Ireland) daily. The last group received both drugs simultaneously, with the exact dosage and same protocol as groups two and three, every 8 h. The medications were prescribed for three weeks. The dosage of medications was based on that of human subjects and also similar to previous studies^19,26–28^.

### Surgical interventions

**Figure 1:**
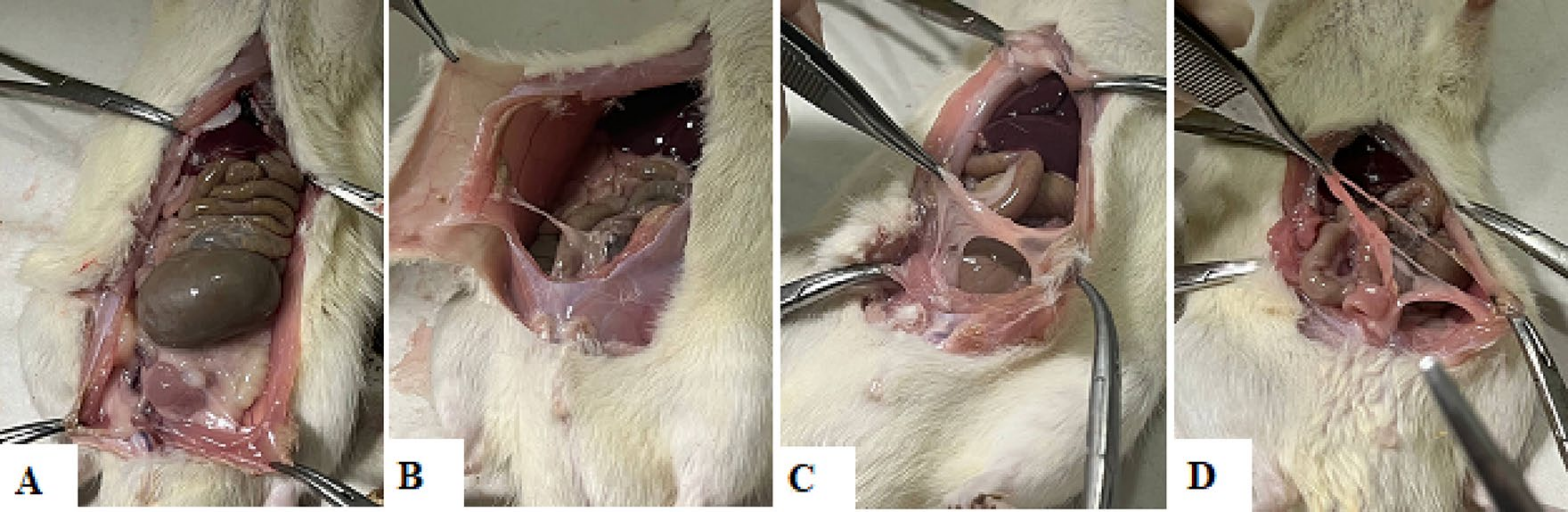
Necropsy pictures from subjects of our experiment, showing the differing grades of adhesion formation; (**A**): No adhesion formation; (**B**): Mild grade 1 adhesions; (**C**): Moderate grade 2 adhesions; (**D**): Moderate to severe adhesion formation.

One week after finishing medication administration, on the 28th day, all rats preoperatively were euthanized with decapitation based on animal guidelines,^29^ before performing laparotomy as in similar studies^30,31^. The surgical procedures were performed under aseptic conditions in a dedicated microsurgical animal operating center. Their abdominal cavities were explored by a single independent surgeon blinded to the subjects’ study groups (Fig. 1). Pathological adhesion formed tissues were fixed in 10% neutral formalin buffer, and was transferred for microscopic evaluation.

### Grading system

The adhesions were assessed and recorded as grades 0–4 according to the Nair classification^32^ (Table 1).

**Table 1:** Degree of adhesion based on Nair classification in rats^32^.

Grade	Description of adhesion band	Remarks
0	Complete absence of adhesions	Insubstantial adhesions
1	Single-band of adhesions, between viscera or from one viscus to the abdominal wall	Insubstantial adhesions
2	Two bands, either between viscera or from viscera to abdominal wall	Substantial adhesions
3	More than two bands, between viscera, or viscera to the abdominal wall, or whole intestines form a mass without adherence to the abdominal wall	Substantial adhesions
4	Viscera is directly adherent to the abdominal wall, irrespective of the number and extent of adhesive bands	Substantial adhesions

### Statistical analysis

The subject’s data were entered into SPSS version 23 (IBM, United States). The count and percentage were calculated for qualitative variables. Comparison between two groups was performed using the Chi-square test or Fisher exact test. Statistical significance was defined as *P* < 0.05.

### Ethical approval and consent to participate

All experimental protocols were approved by the Institutional Animal Care and the Ethics Committee of the Shiraz University of Medical Science (Ethical code: IR.SUMS.MED.REC.1395.S221). Also, the study was carried out in compliance in accordance with the relevant guidelines and also the ARRIVE guidelines.

## Results

Among the 64 rats in our study, 63 were healthy and survived the study period, and were all sacrificed on the 28th day post adhesion induction and after treatment administration. The only mortality in our study was in the combination therapy of the Everolimus and Prednisolone group. The grading of adhesion in each group has been summarized in Table 2

**Table 2:** Comparison of grades of adhesion bands among control group rats with those of rats who received Prednisolone, Everolimus, and combination drug-treated group.

Adhesion		Group n (%); N = 63			
		Control Group; n = 16	Prednisolone; n = 16	Everolimus; n = 16	Combination Therapy; n = 15
Grade	0	0 (0%)	8 (50%)	7 (43.75%)	8 (53.3%)
	1	8 (50%)	3 (18.75%)	4 (25%)	2 (13.3%)
	2	4 (25%)	3 (18.75%)	4 (25%)	2 (13.3%)
	3	1 (6.25%)	2 (12.5%)	1 (6.25%)	0 (0%)
	4	3 (18.75%)	0 (0%)	0 (0%)	3 (20%)
Degree	Insubstantial	8 (50%)	11 (68.75%)	11 (68.75%)	10 (66.6%)
	Substantial	8 (50%)	5 (31.25%)	5 (31.25%)	5 (33.3%)

**Figure 2:**
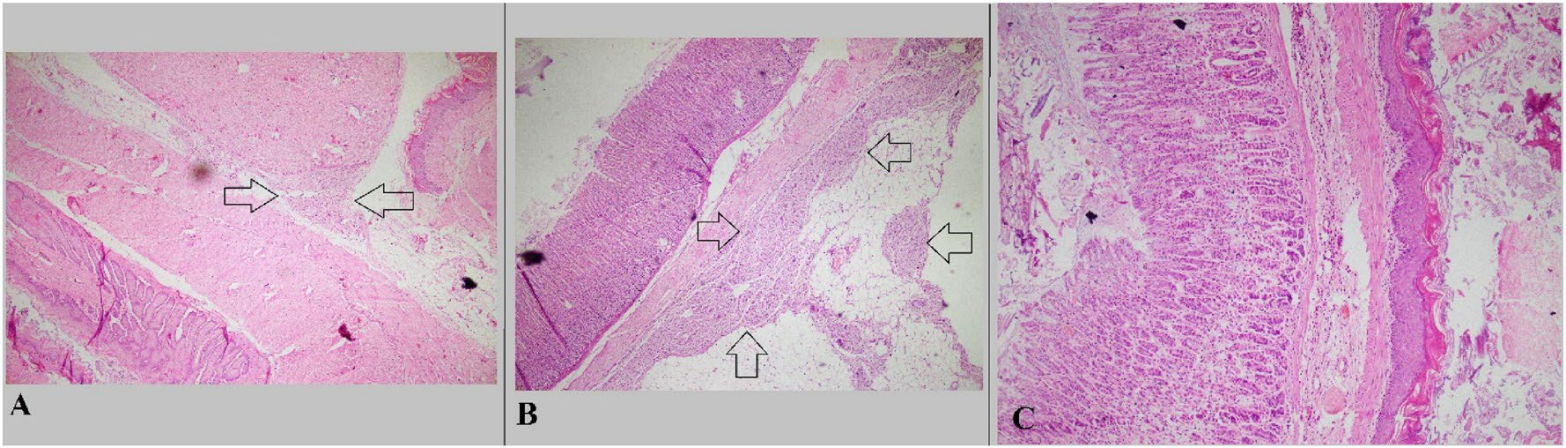
Microscopic sections from cases with significant fibrotic bands; (**A**): Section shows adhesion of esophagus and liver tissue by fibrotic band (Arrow) (Hematoxyline and eosin, × 40); (**B**): Section shows fibrosis and foreign body type reaction in serosa of stomach (Arrow) (Hematoxyline and eosin, × 40); (**C**): Section shows attachment of stomach and esophagus by fibrous band (Hematoxyline and eosin, × 100).

**Figure 3:**
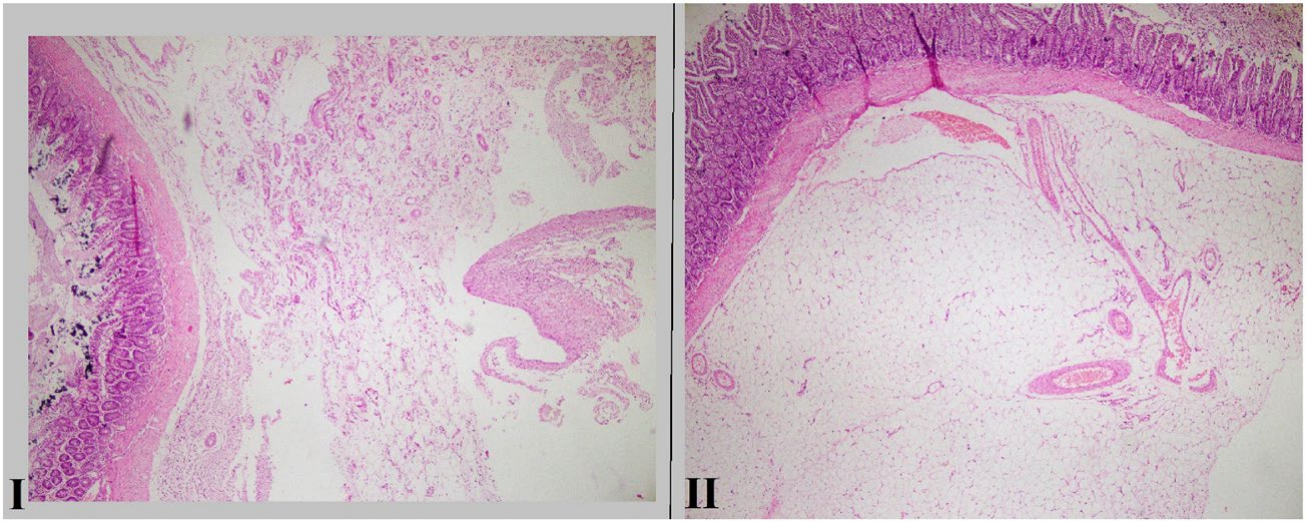
*I:* Microscopic section from small intestine shows mild fibrosis in serosa (Hematoxyline and eosin, × 40); *II*: Microscopic section from small intestine shows no fibrosis in serosa (Hematoxyline and eosin, × 40).

The rats in the control group had extensive adhesions between the abdominal wall and the organs. There were no animals with grade 0 in the control group, and 50% (8/16) of animals in this group had substantial adhesions. However, results showed the rate of substantial adhesion formation in groups receiving Prednisolone, Everolimus, and combination treatment was 31%, 31%, and 31%, respectively. Also, 68.75% (5/11) of the Prednisolone recipients had insubstantial adhesions, the same as Everolimus recipients, while in the combination group, 66.66% (10/15) rats had insubstantial adhesions. Figure 2 displays adhesion formation in control group subjects in our experiment, and Fig. 3 demonstrate a specimen of effective prevention of preventing adhesion formation in treatment groups.

Adhesion bands were categorized based on their degree (Table 1). Based on the fishers’ exact test, there was no significant difference among the groups in our study (*P* = 0.636).

## Discussion

We demonstrated a potent effect of Everolimus as an antiadhesive agent, proliferative inhibitor agent belonging to the origin of macrolide antibiotics^33^ in decreasing viscera adhesion formation in an animal model. A 19% decrease rate of substantial adhesion was observed after the administration of Everolimus compared to the control group. Based on the significant medical and financial burdens following surgical adhesions, prevention or reduction is an essential priority for all surgeons. A wide variety of strategies have been reported through the decades, including surgical and pharmaceutical approaches^10,34^. Everolimus provides a favorable alternative within the current immunosuppressive regimen.

The mechanism of adhesion formation postoperatively arises from a cascade of events in which fibrin plays a critical role^3,35^. Fibrin deposition and activation follow the release of chemical mediators in the adhesion formation cascade, which occurs in peritoneal healing, surgical trauma, or other triggers such as inflammation, infections, chemotherapy, radiation, and malignancy that may cause tissue damage^35–37^. Sartori et al.^38^, regarding impaired fibrinolytic capacity caused by increased plasminogen activator inhibitor type 1 (PAI-1) in renal transplant recipients, suggested that steroid-free immunosuppression like Everolimus is associated with better fibrinolytic behavior, particularly compared to Prednisolone. Their data further confirmed that avoiding long-term steroid therapy is associated with better fibrinolytic capacity. In this regard, Everolimus as a steroid-free agent regulates the fibrinolytic process and can be proposed as a valuable anti-adhesiogenic agent.

The kinetics of the cellular changes in the peritoneal healing process has been extensively reported. It has long been known that particulates, such as talc, cause adhesions of serosal surfaces by stimulating mesothelial cells to secrete chemokines such as IL-15, a growth factor and activator of T cells^39,40^. IL-15 plays a vital role in the immunological cascade leading to the proliferation of activated CD4 cells^41^. Th1 CD4 + ąß cells are crucial for developing intraperitoneal adhesions, and shortly after tissue injury and throughout adhesiogenesis, these activated T-cells become predominant in the abdominopelvic cavity^42,43^. Therefore, several immunosuppressive agents have been proven to reduce postoperative intraperitoneal adhesions in experimental models, such as Tacrolimus^44^, Sirolimus^13,19,45^, mycophenolate mofetil, and cyclosporine^46^. However, it has been formerly demonstrated that vascular toxicity mediated by Calcineurin inhibitors, such as cyclosporine and tacrolimus, might induce endothelial damage leading to impaired fibrinolytic capacity^47^. A study conducted in 2014 showed that consumption of Everolimus in healthy elderly improved protective response after an influenza vaccination as a consequence of expression reduction in programmed cell death-1 receptor on CD8 + and CD4 + T-cells^48^. A more recent study by Guler et al.^49^ showed the significant impact of oral Everolimus effectively reduced adhesion formation compared to Sirolimus. In addition, according to the investigation of Wehling-Henricks et al.^50^, prednisolone can also mediate the reduction of CD4þ T-cells as an effective anti-inflammatory in dystrophic muscle. Our study observed similar effects of Everolimus with those of Prednisolone and a combination regiment of these two in diminishing peritoneal adhesion.

A better understanding of peritoneal adhesion formation at a cellular and molecular level would help to figure out why the combination therapy does not synergize the effect of each single medication. As a result of tissue hypoxia, mesothelial cells secrete chemotactic cytokines and several growth factors dysregulate, such as transforming growth factor-beta 1 (TGFF-β1), PAI-1, interleukin-1 (IL-1)^13,51^. The identification of reliable biomarkers in patients receiving Everolimus or Prednisolone revealed the down-regulation of mentioned molecules^52–55^. Thus, similarity in the mechanism of inhibiting cascade may illuminate the explanation for lack of advantage in combination therapy along with considering side effects of prednisolone, including immune suppressant and the possible drug-drug interactions of medications metabolized by cytochrome P450 (CYP) 3A4 system^56^. Based on our results, the combination therapy demonstrated no significant advantage; therefore, single medication therapy can achieve satisfactory results and also avoid possible drug interactions and side effects while also providing the opportunity to choose the most suitable and appropriate medication regimen for each patient. Even though we did not achieve any statistically significant difference among the groups, the clinical difference cannot be ignored, which can also be achieved statistically in more extensive population studies. In the administration of oral Everolimus, none of the subjects developed adhesion grade 4, while only one case developed a grade 3 adhesion; therefore, its effectiveness in reducing the severity of adhesion formation is prominent. Furthermore, subjects without adhesion were more frequent in rats receiving a combination of Everolimus and Prednisolone than in subjects receiving no treatment. At the same time, the combination group showed no significant advantages in preventing peritoneal adhesions compared to the single therapy groups. Therefore, solus administration of Everolimus could demonstrate an effective antiadhesive generic trait.

Among the other advantages of Everolimus is its antibiotic properties. The intra-abdominal infection produces excessive proinflammatory cytokines that reform peritoneal fibrinolytic activity and eventually induce severe peritoneal adhesion formation. As these factors have been cited as the cause of adhesion formation, Everolimus, through its IL-2- and IL-15-mediated T-cell proliferation inhibition properties can reduce the possibility of adhesion formation^57^.

There have been controversial reports regarding administering steroidal immunosuppressants as anti-adhesiogenic agents. Corticosteroids modify the inflammatory response by decreasing vascular permeability and reducing the liberation of cytokines and chemotactic factors^35^. It has been reported that prescribing methylprednisolone and dexamethasone, aside from administration, can decrease the number of adhesions without influencing the severity of adhesions^17^. Our study demonstrated that oral administration of Prednisolone could effectively reduce the severity and incidence of adhesions compared to the control group. However, the systemic side effects of corticosteroids such as immunocompromised and suspension of wound healing should be considered^58^.

Among the other mechanism of effects of Everolimus is its proliferation signal inhibitor property which is considered unique in the class of immunosuppressive agents due to its anti-oncogenic capacity^33^. The increased risk of post-transplant malignancy can be associated with impaired immune surveillance, which is an unavoidable consequence of the imposed immunosuppressed state of these patients^33,59^. There is some clinical evidence regarding Everolimus concentration-dependent antitumor activity^60,61^ and its efficacy in preventing post-transplant malignancies^62^. Therefore, administration of free steroid protocol like Everolimus may propose a decent safety profile compared to corticosteroids such as Prednisolone. However, further clinical studies with long-term follow-ups are required to support this theory.

Among the limitations of our study is that we evaluated adhesion during an early postoperative period which might not correlate with the degree of adhesion formation that perseveres after longer intervals. However, the point of evaluating adhesion formation in an early postoperative term was related to animal care and the limitations of the model we used. Hence further studies are demanded to investigate the influence of immunosuppression on adhesion formation after longer intervals associated with the clinical practice. Also, the systemic side effects of the studied drugs should be evaluated, and their adhesion prevention properties should be weighed against their consequences. Furthermore, Prednisolone and Everolimus were only available in oral form in our country, while studies addressing other routes of administration are justified. Finally, further studies in human subjects, along with histopathological evaluations, are needed to confirm the efficacy of these agents for peritoneal adhesions prevention. Furthermore, the immune cellular pathway and related cytokines and chemokines and its’ direct role in the pathogenesis of adhesion formation were not assessed in our study, which warrants further molecular and immunocytological studies in this regard.

## Conclusion

Everolimus demonstrated satisfactory results in reducing the rates of induced peritoneal adhesion in an experimental model, similar to those of Prednisolone and superior to a combination regime. Further clinical studies evaluating Everolimuses' antiadhesive characteristics, along with its anti-neoplastic and antibiotic properties and possible adverse effects can provide valuable evidence for the application of this medication in clinical practice and guidelines.

## Data Availability

All data regarding this study has been reported in the manuscript. Please contact the corresponding author if interested in any further information.
